# Fostering Engagement With Health and Housing Innovation: Development of Participant Personas in a Social Housing Cohort

**DOI:** 10.2196/25037

**Published:** 2021-02-16

**Authors:** Andrew James Williams, Tamaryn Menneer, Mansi Sidana, Tim Walker, Kath Maguire, Markus Mueller, Cheryl Paterson, Michael Leyshon, Catherine Leyshon, Emma Seymour, Zoë Howard, Emma Bland, Karyn Morrissey, Timothy J Taylor

**Affiliations:** 1 School of Medicine University of St Andrews St Andrews, Fife United Kingdom; 2 European Centre for Environment and Human Health University of Exeter Truro, Cornwall United Kingdom; 3 Environment and Sustainability Institute University of Exeter Penryn, Cornwall United Kingdom; 4 College of Engineering, Mathematics and Physical Sciences University of Exeter Exeter United Kingdom; 5 Centre for Geography and Environmental Science University of Exeter Penryn, Cornwall United Kingdom

**Keywords:** user-centered design, community, social network analysis, United Kingdom, mobile phone

## Abstract

**Background:**

Personas, based on customer or population data, are widely used to inform design decisions in the commercial sector. The variety of methods available means that personas can be produced from projects of different types and scale.

**Objective:**

This study aims to experiment with the use of personas that bring together data from a survey, household air measurements and electricity usage sensors, and an interview within a research and innovation project, with the aim of supporting eHealth and eWell-being product, process, and service development through broadening the engagement with and understanding of the data about the local community.

**Methods:**

The project participants were social housing residents (adults only) living in central Cornwall, a rural unitary authority in the United Kingdom. A total of 329 households were recruited between September 2017 and November 2018, with 235 (71.4%) providing complete baseline survey data on demographics, socioeconomic position, household composition, home environment, technology ownership, pet ownership, smoking, social cohesion, volunteering, caring, mental well-being, physical and mental health–related quality of life, and activity. K-prototype cluster analysis was used to identify 8 clusters among the baseline survey responses. The sensor and interview data were subsequently analyzed by cluster and the insights from all 3 data sources were brought together to produce the personas, known as the Smartline Archetypes.

**Results:**

The Smartline Archetypes proved to be an engaging way of presenting data, accessible to a broader group of stakeholders than those who accessed the raw anonymized data, thereby providing a vehicle for greater research engagement, innovation, and impact.

**Conclusions:**

Through the adoption of a tool widely used in practice, research projects could generate greater policy and practical impact, while also becoming more transparent and open to the public.

## Introduction

### History of Personas

In 1999, software developer Alan Cooper [1] published the book “The Inmates are Running the Asylum” in which he advocated “user-centered design.” To focus the design of software or any other product on the intended user, Cooper suggested using “personas” [1-5]. Since then, personas have been applied in a wide variety of fields where systems, services, or products are being designed for human use. Such applications include health service design [3,6,7], eHealth services [2,6,8-13], and health behavior change [14-17].

There is a long history of typology in the social sciences, whether seeking to identify types of individuals, organizations, or societies. Both Plato and Aristotle considered there to be forms that were not specific to any person or entity but representing some fundamental collective characteristics, which are seen as the origins of the concept of archetypes [18,19]. Typologies and categorical groups have been useful in the development of the understanding of various aspects of society, such as politics, history, and development [20,21]. Jung developed the earlier ideas around archetypes in the field of psychology as innate and universal primordial ideas (prototypes), which were useful for interpreting behaviors and actions [22]. Ernest Dichter later applied Jung’s archetypes to advertising and marketing [23].

Possibly, the most well-known use of a persona in health care services in the United Kingdom was Torbay and South Devon Health and Care National Health Service Trust’s “Mrs Smith,” a persona of an older woman created to support the integration of health and social care services [24-26]. Most recently, methods for persona development have begun to be applied in research projects to support the understanding of the complex system of social determinants of health [27-29]. In addition, the expansion in the forms and amounts of data collected make it necessary for data producers and data holders to present data in formats that are more accessible while maintaining participant confidentiality.

### Persona Development Methods

Two significant challenges in developing personas are avoiding harmful stereotypes and achieving a balance between making the personas relatable so that they are engaging but avoiding being so specific that they do not relate to a large enough group of people (customer base) [5,30]. The process for developing personas typically comprises a number of steps, starting with identifying basic details of the personas, such as demographics, and subsequently adding layers of detail until a sufficiently life-like and relatable character is formed [3,16,30]. The types of details required for the persona are selected to fit the purpose for which the personas are being designed. For example, the designer of a new magazine would want details about the interests and lifestyle choices of the persona, whereas the designer of a health service would want to know the persona’s health state and treatment preferences. The approaches taken to this process vary from those based purely on anecdotes or experiences and are therefore more prone to stereotyping [2,7,14,31], through to those based on the objective analysis of data reducing the risk of stereotyping within the personas [3,10,12,13,17,20,27,32,33]. Most people advocate a mixed methods approach using both objective and subjective data to avoid stereotypes and overly specific personas [3,9,10,16,17]. Recently, those who will use the eventual product, process, or service have become involved in the creation of the personas to be used in the development process [2,6,8,34].

The common quantitative methods often applied to persona development are factor analysis [33], latent class analysis [27], k-means cluster analysis [12,13,17], and hierarchical cluster analysis [3,10,32]. The different quantitative methods relate to whether the fundamental characteristics of the clusters are observable or latent hidden attributes. Hagenaars and Halman [20] and Floyd et al [4] have critiqued and compared the various methods based on their statistical properties; however, it is likely that the most appropriate methods for creating personas will depend on the specific scenario and how they will be used.

Regardless of specific methods, there is agreement about the value of the personas in terms of provoking empathy, interest, and understanding; providing grounding and personalization; and supporting better product, process, and service development [2,5,27,28,31,33]. Pruitt and Grudin [5] wrote that personas “provide a conduit for conveying a broad range of qualitative and quantitative data, and focus attention on aspects of design and use that other methods do not.”

The aim of this study was to experiment with the use of personas within a research and innovation project to support product, process, and service development through broadening the engagement with and understanding of the data about the local community. In this paper, we outline the innovative mixed methods process we have applied to generating personas of social housing residents and the uses to which these have been put to date. Although the initial process was an established technique for data-driven persona development [12,13,17,33], the qualitative methods and addition of sensor data are more novel. Holden et al [3] advocated for the combination of quantitative and qualitative data “to produce richer, contextualized descriptions of personas” (p. 165), but admitted that they were only minimally able to incorporate qualitative data into their personas. Consequently, the significant incorporation of qualitative data into the final Smartline Archetypes is a novel contribution of this study. In addition, it was hoped that the personas would support participant engagement with their own data and increase the transparency of the project. Subsequently, the derivation of the personas from individual data, but representing groups of people through a fictional life-like persona, can maintain privacy while increasing accessibility to the data.

### The Smartline Project

The Smartline project is a European Regional Development Fund–funded research and innovation project focused on household and community health and well-being. Its purpose is to develop the eHealth and eWell-being sector in Cornwall and the Isles of Scilly in the United Kingdom [35] through collaboration between academia and business, specifically the University of Exeter, Coastline Housing (a social housing provider), Volunteer Cornwall, and Cornwall Council [36]. Cornwall is a county in the southwest of England; it only borders one other county, with the other border being the coastline. Considered a rural county, the settlements include many dispersed small villages and towns with populations up to approximately 25,000. Previous studies describing other aspects of the project have examined the associations between health and mold [37] and social cohesion [38] among the participants.

In the United Kingdom, 99.61% (2,738,980/2,718,435) of businesses are small- and medium-sized enterprises (SMEs), with fewer than 250 employees, and in Cornwall, many are microenterprises, with fewer than 10 employees [36,39,40]. Although Smartline had the opportunity to share consented and anonymized data with project partners and local enterprises, such organizations and businesses are unlikely to have the capacity or data science skills required to interact with large quantitative data sets. Therefore, it was necessary for Smartline to present data in formats that are more accessible. A Smartline Knowledge Exchange Officer (ES) had used personas in market research settings and recognized their potential to address data accessibility for SMEs in the region.

The participants were adults (older than 18 years) recruited from among Coastline Housing residents in the towns of Camborne and Redruth and the villages of Illogan and Pool. Together, these form the largest conurbation in Cornwall, with a population of 47,500 in the last census in 2011 [41,42]. Moreover, these locations were selected because they provided a high concentration of Coastline Homes needed to address the project’s focus on communities and individual households. Coastline Housing undertook participant recruitment street-by-street between September 2017 and November 2018. In total, 649 households were approached; 329 were recruited into the project and completed baseline data collection (329/649, 50.7% response rate).

**Figure 1:**
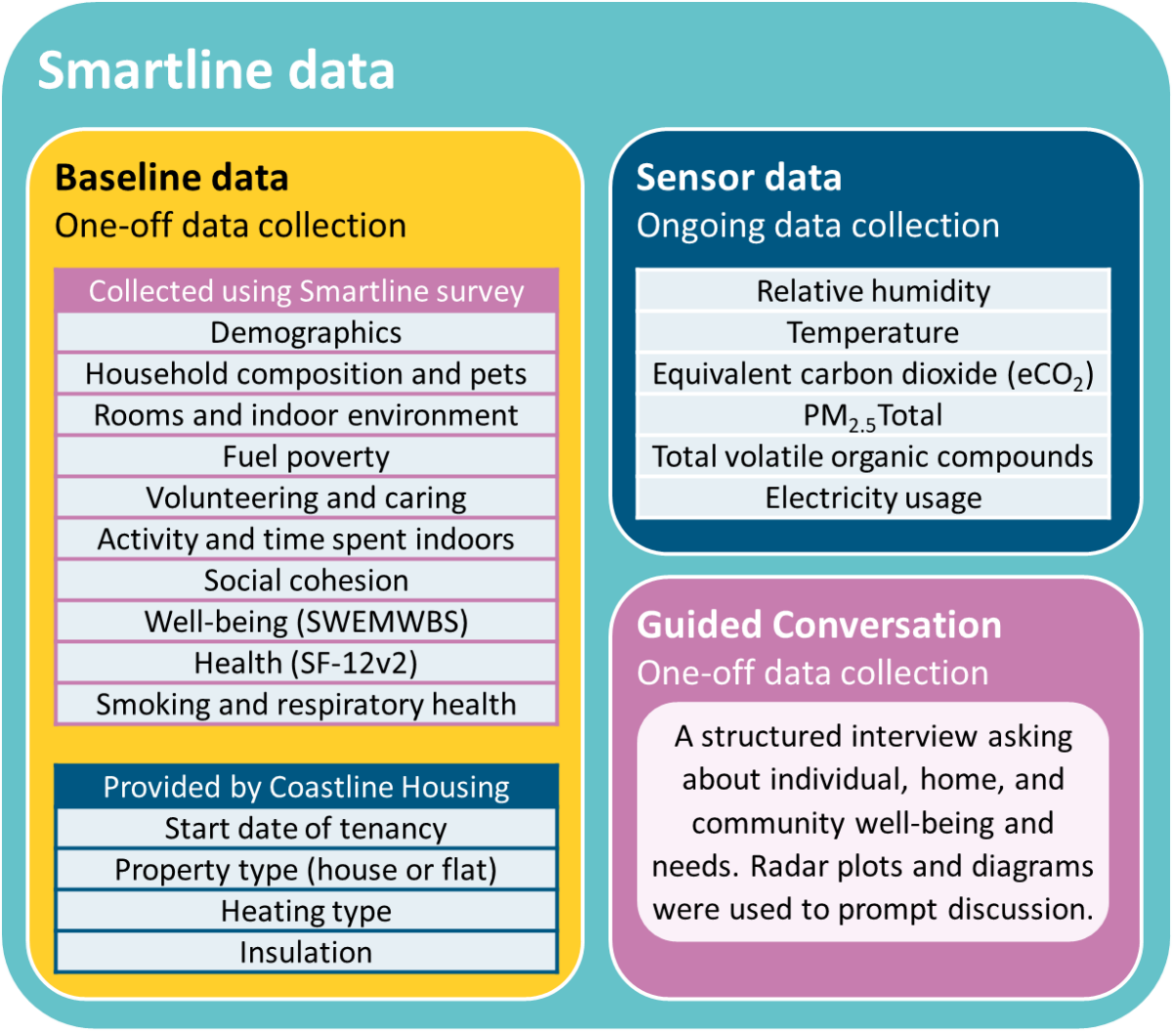
Smartline project data sets. eCO_2_: equivalent carbon dioxide; PM_2.5_: atmospheric particulate matter that have a diameter of less than 2.5 m; SWEMWBS: Short Warwick Edinburgh Mental Wellbeing scale.

The project collected a variety of data (Figure 1), using a face-to-face survey, environmental and electricity usage sensors, and a structured interview called a Guided Conversation [43,44]. The project was reviewed by the University of Exeter Research Ethics Committee, and all participants provided written informed consent. All participants needed to consent to participate in the survey and to have sensors installed to join the project, but participation in the Guided Conversation was not a requirement. The surveys and Guided Conversations took place at a convenient time in the participants’ homes with 2 researchers present. Sensor data measurements were recorded approximately every 3-5 min. The data were collected to stimulate innovation within the project in partnership with businesses and voluntary organizations working with the project. The personas were developed using the various data collected throughout the project to stimulate further innovation.

## Methods

### Persona Development Process

**Figure 2:**
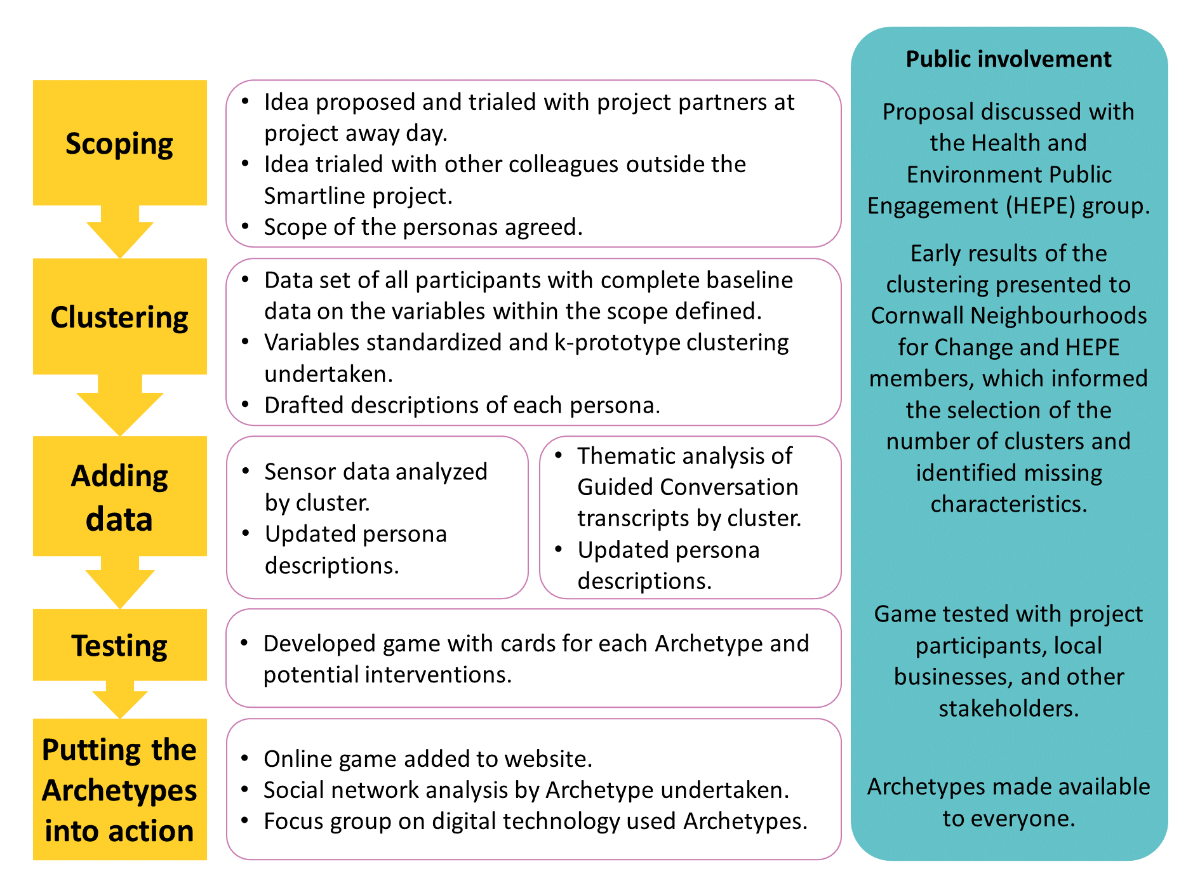
Persona development method for the Smartline Archetypes. HEPE: Health and Environment Public Engagement group.

The process used to develop the personas, illustrated in Figure 2, followed the common steps of initially specifying some basic details about each persona using the baseline data and then layering on further details (the sensor and Guided Conversation data) until life-like individuals were created [3,16,30]. Smartline participants and broader public groups were involved throughout the process to ensure that the final personas were acceptable, accessible, and true to people’s experience [16]. Initially, the idea was presented to a number of groups to test whether it was considered worth pursuing and to define the scope of the personas. The next step was to undertake a cluster analysis of the survey data.

### k-Prototype Cluster Analysis

Cluster analysis (also known as segmentation or taxonomy analysis) is a statistical method for interpreting a large data set by grouping the records into clusters. Each record has values for a set of variables (eg, gender, age, socioeconomic status). Cluster centers are randomly generated, each being a set of variable values. Records are assigned to the most similar cluster center, and cluster membership is iteratively updated to minimize the difference between records and their cluster center, based on the variable values. Records in the resulting clusters are generally more similar to one another than to records in other clusters [45]. The k-prototype approach, similar to other clustering techniques, is an unsupervised machine learning method. Developed from the k-means and k-modes methods, the k-prototype method can handle both continuous and categorical data [45,46]. The method minimizes the Euclidean distance for numerical factors, as in k-means clustering, and uses the number of mismatches between data points for the categorical variables [45].

The baseline data used for the cluster analysis included variables related to demographics, socioeconomic position, household composition, experience of home environment (comfort, mold, and fuel poverty), technology ownership, pet ownership, smoking, social cohesion, volunteering, caring, mental well-being (Short Warwick-Edinburgh Mental Wellbeing Score [47-50]), physical and mental health–related quality of life (12-item Short Form Health Survey, version 2 [51,52]), and activity (Figure 1). All variables included in the cluster analysis were quantitative data from the survey responses or the Coastline Housing data. The inclusion of these variables would allow the personas to reflect multiple aspects of the participants’ lives and demonstrate the breadth of data held by the project. List-wise deletion based on the 329 selected variables left 235 (71.4%) participants on whom to conduct the cluster analysis. To account for the various scales of each variable, the data were standardized using *z*-scores before the clustering analysis. The k-prototype clustering was performed in R using the package “clustMixType” [53,54], with participants assigned to the cluster that most closely matched their characteristics.

It is necessary to specify the number of clusters to be calculated when conducting k-prototype analysis. In the literature, there are both analytical and pragmatic techniques for identifying the appropriate number of clusters [13,17]. With the intention that the personas would be accessible to the public, it was important for us to triangulate these data-driven decisions with community-focused perspectives. To those ends, both the techniques to be used and the potential granularity of the clusters were discussed with 2 groups of community partners: Health and Environment Public Engagement group and Cornwall Neighbourhoods for Change. Similarity within each cluster increases with the number of clusters. The optimum number of clusters is often chosen to be the number at which little is gained by adding more clusters. This method is known as the elbow method, using a plot as presented in Multimedia Appendix 1. However, we found no clear analytical evidence for selecting a given number of clusters over another number. In addition, feedback on granularity from community partners and on business requirements from Smartline’s Knowledge Exchange Officer (ES) suggested that a maximum of 8 clusters would be appropriate. Pragmatically, the 8 clusters were also sufficiently populated to capture a generalization across multiple people (average of 29 participants per cluster). Patterns within the summary statistics of the variables within each cluster were examined to characterize each Smartline Archetype. All the data from the survey were included in the characterization of the Smartline Archetypes, not just those variables included in the cluster analysis.

### Sensor Data

Using the unique participant identifiers, the data from environmental and electricity usage sensors for each home were allocated into each of the 8 clusters. The mean sensor data readings were calculated for each household over all the readings taken in 2019. In line with the choice to use the term archetype, we anticipated that the variation within the sensor outcomes of each Smartline Archetype would be of interest, for example, to compare a high- and low-electricity user of the same Smartline Archetype. Hence, the Smartline Archetypes were integrated into the project data-sharing platform.

### Qualitative Analysis

A subsample of 62 semistructured qualitative interviews (known as “Guided Conversations”) were conducted. Participants were selected via nonprobability sampling out of a total sample of 329 participants. The interviews lasted for an average of 45 min and were conducted face to face by 2 researchers between November 2017 and May 2018. The responses were recorded directly onto a script by the assigned note taker and transcribed to a database after the interview. Participants were not paid, and interviews took place during a time that was most convenient for the participant. The purpose of these interviews was to identify well-being priorities and then develop an achievable action plan with the participant. They were structured around the 3 themes of well-being, home, and community under which there were a series of prompts. These themes and prompts were arrived at through a co-design process involving all project partners. The interview guide was piloted with 4 voluntary and community sector organizations and 5 Coastline Housing tenants and adjustments made.

Using unique study identifiers, the transcripts of each interview were allocated to the clusters. There was an uneven spread of interview data across Smartline Archetypes: #1, 6 interviews; #2, 3 interviews; #3, 3 interviews; # 4, 4 interviews; # 5, 6 interviews; #6, 4 interviews; #7, 10 interviews; and #8, 5 interviews. Due to the list-wise deletion of survey records, not everyone who was interviewed was allocated to a Smartline Archetype.

An interdisciplinary team of 10 researchers conducted a 5-step collaborative data analysis exercise with the interview transcripts. Using multiple coders increases the rigor in a qualitative analysis by drawing upon diverse perspectives and counteracting individual biases in the coding process as interpretations and assumptions are placed in the plain view of the group [55,56]. This method also allowed us to reasonably manage the large data set [55]. In this study, we chose to conduct the coding manually for the following two reasons. First, many of the interdisciplinary teams were unfamiliar with qualitative analysis software, and therefore, time-intensive training would be required [57]. Second, the marking up, sorting, and reorganizing of transcripts was deemed a manageable task given the 10-strong team of researchers.

Thematic analysis of interview transcripts involved a systematic 5-step process (Textbox 1). Through this exercise, the team produced a codebook that was transparently documented and justified the analytical decisions [55,58]. The outputs were additions or adaptations to each Smartline Archetype description and a 3-point list of headline descriptors. The team was split into 5 pairs, each of whom coded 2 Smartline Archetypes.

**Five-step thematic analysis process.**

Step 1: Data familiarization and identification of significant topics

- Each pair familiarized themselves with their Smartline Archetypes transcripts and examined the graphs, which illustrated the satisfaction scores from the radar plots for each of the interview topics. The graphs facilitated quick identification of the highest and lowest scoring topics within and between each Smartline Archetype. The output of this iterative process was the identification of a significant topic or topics for each of the Smartline Archetypes.

Step 2: Open coding and subtheme development

- Open coding is the process of identifying discrete concepts and patterns in the data [59]. The team employed this process on the significant topics, identified in step 1, for each Smartline Archetype.

Step 3: Axial coding and theme identification and triangulation

- Axial coding is the dynamic and creative process of identifying connections between patterns in the data [57,59]. The team used this process in reference to the Smartline Archetype characteristics produced by the cluster analysis and the open codes. This iterative process enabled points of triangulation to be identified between the quantitatively derived characteristics and the interview data. The output from this step was the identification of a 3-point list of headline descriptors for each Smartline Archetype.

Step 4: Pull exemplar quotes from transcripts

- Quotes that exemplified the significant theme were then pulled from transcripts and added to the code book.

Step 5: Write summary sentence

- The final step was to write sentences that summarized the themes identified and insert them into the Smartline Archetype description.

### Testing

To test if the Smartline Archetypes were acceptable, accessible, and true to people’s experience [16], we produced a “serious game,” that is, one used for more than just entertainment [60]. Each Smartline Archetype was allocated a name and a cartoon image, presented as “Top Trumps” cards. The game, which involved matching attributes to characters, was played at community events involving project participants and events attended by businesses. The feedback from participants supported the use of the Smartline Archetypes, and most people found at least one Archetype that they could relate to themselves or a neighbor. The cards also prompted conversations with participants around the support, services, or products that might be useful to that Smartline Archetype. Providing people with a character that is similar but distinct from themselves has previously been used to prompt reflection and potential behavior change by Wyatt et al [61] and Brown et al [62].

### Putting the Archetypes Into Action

To date, the Archetypes have been used in three different ways. First, an updated version of the persona card game was turned into a “game” that can be played on the Smartline website [63]. Second, the personas have also been used to facilitate focus groups with participants to gather views about behaviors and attitudes toward digital technology [64]. Third, the Smartline Archetypes have also been explored as part of a social network analysis of the participants (Stevens et al, unpublished data, 2021).

## Results

### The Smartline Archetypes

The 8 clusters identified by the k-prototype analysis of the baseline survey data are summarized in Table 1. Two-thirds of Smartline participants were female, and their ethnic diversity reflects that of Cornwall, with only 3.9% (10/256) from an ethnic minority. The Smartline Archetypes reflected these demographics. However, public engagement with community partners identified that it was important to include some diversity among the Smartline Archetypes. Therefore, the 4 Archetypes with the lowest likelihood of being female were designated male, and the Archetype with the highest proportion of ethnic minority participants was presented as being from an ethnic minority (Archetype #6).

**Table 1:** Summary characteristics of the 8 clusters identified by the k-prototype analysis of the baseline survey data.

Characteristic	Cluster							
	1	2	3	4	5	6	7	8
Participants, n (%)	24 (10.2)	28 (11.9)	18 (7.7)	31 (13.2)	28 (11.9)	23 (9.8)	54 (23.0)	29 (12.3)
Female, n (%)	16 (67)	17 (601)	14 (78)	23 (74)	17 (61)	15 (65)	38 (70)	21 (72)
Age (years), median	61.0	67.5	52.0	34.0	65.5	66.0	55.0	63.0
National identity, mode	British	British	Cornish	Cornish	Cornish	British	Cornish	Cornish
Ethic minority, n (%)	0 (0)	0 (0)	0 (0)	0 (0)	1 (4)	2 (11)	1 (2)	0 (0)
Employed, n (%)	10 (42)	3 (11)	3 (17)	11 (35)	5 (18)	2 (9)	4 (7)	8 (28)
Retired, n (%)	6 (25)	18 (64)	4 (22)	0 (0)	18 (64)	13 (57)	14 (26)	14 (48)
IMD^a^ 10% most deprived, n (%)	16 (67)	11 (39)	9 (50)	17 (55)	19 (68)	12 (52)	26 (48)	14 (48)
Urban, n (%)	22 (92)	27 (96)	15 (83)	28 (90)	28 (100)	21 (91)	49 (91)	24 (83)
Household size, range	1-3	1-2	1-3	2-4	1-2	1-3	1-2	1-3
Internet access	Yes	No	Yes	Yes	Yes	Yes	No	Yes
Smart meters	No	No	No	No	No	No	No	Yes
Pets (mode)	Cats	Dogs	Cats	Cats	Cats	Dogs	Dogs	Other
Fuel poverty	No	No	Yes	No	No	No	Yes	No
Mold	No	No	Yes	Yes	No	No	Yes	Yes
Smoker	No	Yes	No	Yes	No	Yes	No	No
Volunteering	No	No	Yes	No	Yes	Yes	Yes	No
Physical health	Poor	Poor	Good	Good	Poor	Good	Poor	Good
Mental health	Average	Average	Poor	Average	Good	Good	Poor	Good
Physically active	No	No	No	Yes	Yes	Yes	No	Yes

^a^IMD: index of multiple deprivation.

**Figure 3:**
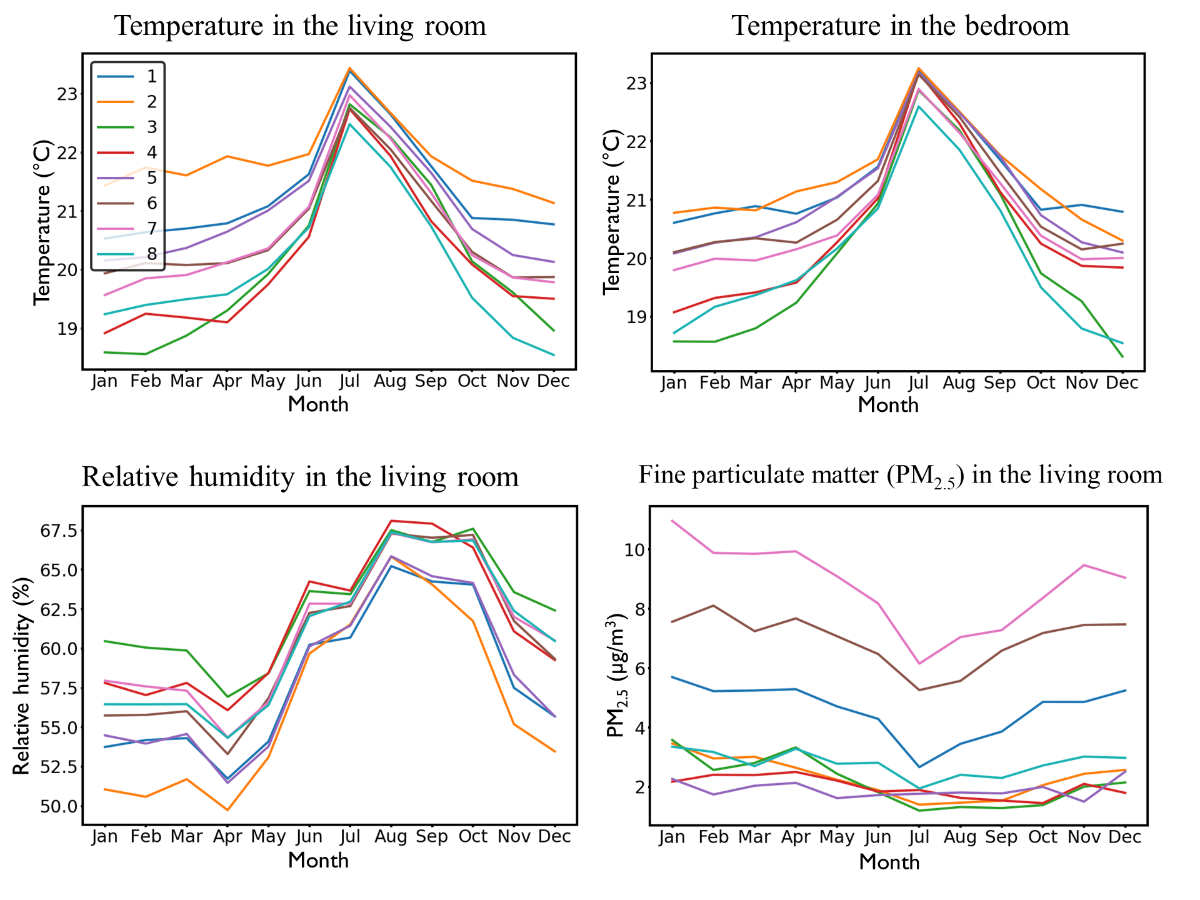
Smartline sensor data monthly means for the archetypes across 1 year. PM_2.5_: atmospheric particulate matter that have a diameter of less than 2.5 μm.

Sensor data types are presented in Figure 1. Mean values were taken over 2019 for each data type and each household. Means were compared across Smartline Archetypes using a separate one-way analysis of variance (ANOVA) for each sensor data type, with the Archetype as the between-participants factor with 8 levels. Significant effects of the Archetype were investigated using the Tukey post hoc test for pairwise comparisons of Smartline Archetypes. There was no significant effect of the Archetype on relative humidity in the bedroom (*F*_7,160_=1.382; *P*=.22; *η*^2^=0.057), PM_2.5_ (atmospheric particulate matter that have a diameter of less than 2.5 μm: *F*_7,115_=1.263; *P*=.28; *η*^2^=0.071), equivalent carbon dioxide (*F*_7,84_=1.246; *P*=.29; *η*^2^=0.094), and electricity usage (*F*_7,75_=0.885; *P*=.52; *η*^2^=0.076). There was a trend toward significance for temperature in the bedroom (*F*_7,160_=1.932; *P*=.07; *η*^2^=0.078) and for relative humidity in the living room (*F*_7,153_=2.024; *P*=.06; *η*^2^=0.085). Temperature in the living room differed across Archetypes (*F*_7,153_=2.380; *P*=.02; *η*^2^=0.098), with higher temperature in Smartline Archetype #2 “David Hartley” than Smartline Archetype #8 “Cathy Johnson” (*P*=.03). The sensor data did not reveal many additional insights about the Smartline Archetypes. However, 4 examples of the sensor data are shown in Figure 3, including those measures with the greatest differences across Archetypes. To illustrate the potential of the sensor data, monthly means were plotted. The temperature sensor data appeared to be consistent with participants who reported issues with temperature in the survey, whereas those Archetypes with higher PM_2.5_ did not seem to be consistent with those living near roads, smoking, or keeping their windows closed. It was clear that there was more variation in the internal environment in winter than in summer.

The qualitative analysis of the Guided Conversations was consistent with the findings of the quantitative cluster analysis. Through the qualitative analysis, it was possible to add depth and explain the features from the survey data. Only within Archetype #7 “Sarah Jones” did triangulating the qualitative and quantitative data prove challenging. This was the largest of the Smartline Archetypes, with more than 20% (54/235, 23.0%) of the participants included in the clustering analysis and 10 Guided Conversation transcripts. Although the quantitative approach clustered these individuals, the Guided Conversation data revealed a variety of circumstances within this Archetype. The people in Archetype #7 experienced a number of complicated circumstances around health, finances, and caring responsibilities for family members. This scenario demonstrates that although the reported data can be similar, there can be significant differences in experience that could be missed without the richness of qualitative data or user engagement. The final descriptions of each of the Smartline Archetypes bringing together the baseline survey data, household sensor, and Guided Conversation data are provided in Table 2.

**Table 2:** The Smartline Archetypes (the percentages are the percentage of the 235 participants in each archetype).

Archetype			Description
**Archetype #1—“Jack Brown,” Male, 59 years old, 10.2% (n=24)**			
	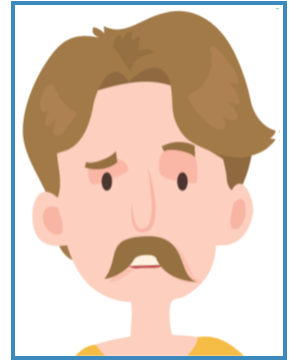	Resourceful—Competent—Capable Jack works for a building merchant and lives with his wife and cat in one of the 10% most deprived neighborhoods in England. They have access to the internet and own quite a few pieces of technology. His health and well-being are about average and he is fairly active. He does not think that mold is affecting their health, despite having mold in the bathroom and his wife having respiratory symptoms. They help out their neighbors. He takes pride in being self-reliant but also knows whom to ask for help and is willing to do this. He feels competent and confident in completing household and “Do-it-Yourself” tasks, even those tasks that he does not necessarily enjoy.	
**Archetype #2—“David Hartley,” Male, 65 years old, 11.9% (n=28)**			
	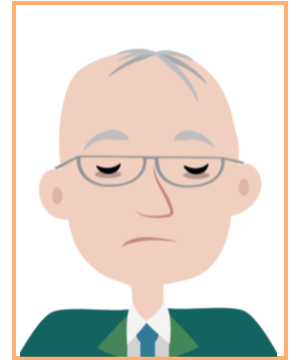	Poor physical health—Insular—Inhibited aspirations David retired early owing to poor physical health. He lives alone and is a moderate smoker. He spends most of his time indoors and does not do any volunteering. He does not own much technology and does not have access to the internet. He has chronic obstructive pulmonary disease and spends most of his time sitting. His mental health is better than his physical health, which affects many aspects of his life, from exercise and recreation to occupation and learning, and their associated aspirations. He is somewhat insular by choice, although he is happy with where he lives. He does not have any mold in the home.	
**Archetype #3—”Mandy Green,” Female, 55 years old, 7.7% (n=18)**			
	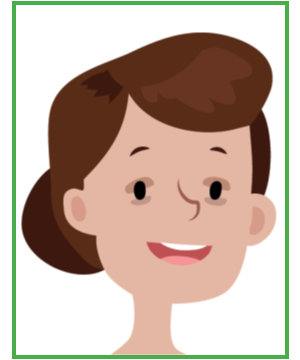	Frequent walker—Well connected—Strong sense of belonging Mandy lives with her daughter who is at college and some cats in a 3-bedroom house in a rural area. She is a self-employed cleaner. She owns a tablet computer and a smart watch. To save money on heating, Mandy only heats certain rooms and avoids opening windows. Mandy spends a lot of time indoors at the weekends but has never smoked. Her physical health is good, but her mental health is poor. Mandy has a strong sense of belonging to her community. She takes walks frequently, including to get to her cleaning jobs. However, reports of crime in the area undermine her sense of safety.	
**Archetype #4—“Jennie Fryer,” Female, 37 years old, 13.2% (n=31)**			
	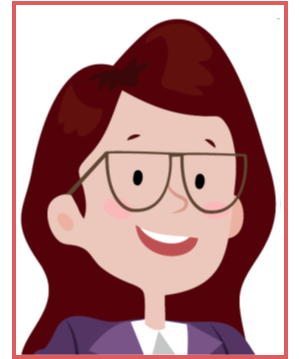	Driven—Resourceful—Informed Jennie lives with her partner and infant child in a 3-bedroom home. While bringing up their child, Jennie is undertaking a National Vocational Qualification course at college. She and her partner smoke, but they both try not to smoke indoors. She owns a smartphone and keeps fish. They have mold in the bathroom and bedrooms, which she is concerned is affecting the family’s health; however, they only heat specific rooms. Jennie wants to work, but the jobs available locally do not fit with her family commitments, skills, and training. She is resourceful and well connected, knowing how to obtain information if she has an issue. She is worried about the lack of parking in the neighborhood.	
**Archetype #5—“Fred Jones,” Male, 65 years old, 11.9% (n=28)**			
	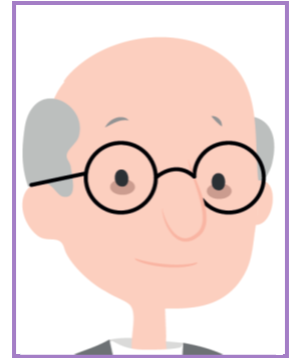	Self-reliant—Happy and active—Reflective Fred is a retired taxi driver living with his wife and a dog in a 1-bedroom house in one of the 10% most deprived areas in England. He owns a smart watch and a laptop. He feels that their home is adequately heated but uses a dehumidifier as they have some mold. Fred and his wife are fairly self-reliant, happy, and active, but reflective as they worry for the future of community and family with regard to community spirit and jobs. He volunteers formally as well as helps out neighbors, as he has a strong sense of social cohesion.	
**Archetype #6—“Raj Singh,” Male, 60 years old, 9.8% (n=23)**			
	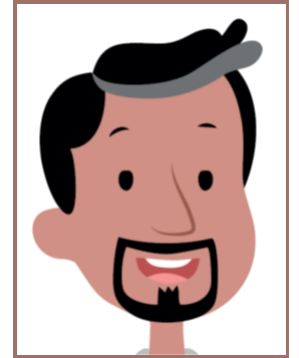	Active community member—Disappointed by local services—Resilient but in pain Raj lives with his adult son and is looking for work in a factory. Although they heat all the rooms in their home, he does not think the home is adequately heated. However, they do not experience much mold. He and his son have quite a few technological devices. Raj smokes and gets short of breath, but otherwise his mental and physical health and well-being are fairly good. He helps out in his community, as he is interested in the standard of services available. Raj is more focused on the community and the services available than his indoor environment.	
**Archetype #7—“Sarah Jones,” Female, 50 years old, 23.0% (n=54)**			
	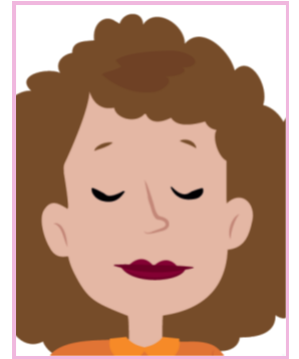	Financial difficulties—Isolated—Caring responsibilities and disability Sarah used to work, but she cannot work now because of a long-term condition. She has some caring responsibilities for an older parent. Sarah has concerns about smart technology and privacy, so she only has a simple mobile phone, which makes it difficult for her to access some services that require internet access. Her home smells moldy, and she is worried about how this is affecting her health. But Sarah does not go outside much and avoids opening window to save heat. She does some volunteering at a charity shop but reports low social cohesion. Sarah is frustrated by her declining mental and physical health, and her limited finances.	
**Archetype #8—“Cathy Johnson,” Female, 61 years old, 12.3% (n=29)**			
	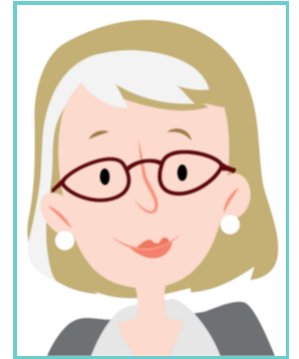	Content on own or with their small group of friends—Frequent walker—Enjoys mentally stimulating activities Cathy is retired and lives in a village with her adult daughter. Cathy has upgraded to smart gas and electricity meters. Her daughter keeps a snake. Cathy dries her clothes indoors and is not concerned about the cost of heating. She does not do any volunteering but has caring responsibilities toward her daughter. Cathy’s hobbies are crosswords, reading, and knitting. She is fairly insular with a close-knit group of friends. Her physical and mental health are good and have been improving. She is fairly busy at home so does not have much interest in the community.	

### Putting the Archetypes Into Action

Having tested the Smartline Archetypes using the “Top Trumps” style cards with a number of audiences, including the project participants themselves, the Archetypes have been put into action. The web-based version of the card game was only launched in summer 2020; therefore, it is still being evaluated. Within the digital technology focus group, participants were asked to decide whether a given Archetype would like to engage with technology and, if so, what kind. Participants’ comments suggested that they felt more able to talk about themselves than the Archetypes, but the Archetypes did provide a conversation facilitator and allowed participants to avoid a more personal perspective as desired. The social networks (ego networks) of the Smartline participants who participated in this study are illustrated in Multimedia Appendix 2, with the Smartline Archetype of the participants denoted by color. An ANOVA analysis of the social network ties by Smartline Archetype identified a statistically significant difference in the number of ties reported by Archetype, with Archetype #5, “Fred Jones,” reporting an average of 12 ties, while the others reported around 4-7 ties. Such information could be useful in community development or spreading health messages, through the identification of those who might spread messages well, or those who are disconnected and might need targeted messages. Various and ongoing engagements of broader stakeholders with the Smartline Archetypes have continued to confirm their validity, and the Archetypes have proved to be an engaging tool for discussions about the project and data. A number of small and microenterprises who had not engaged with the project data before engaged with the Smartline Archetypes, learning about the project participants and the prompting ideas related to their business. The number of Smartline Archetypes and the wealth of information known about each one means that specific Archetypes or specific details can be selected depending on the topic, product, process, or service being discussed.

## Discussion

### Principal Findings and Implications

The process of developing personas to inform product, process, and service development has been widely adopted across multiple sectors including health care [1-3,6-17,24-26]. Qualitative and quantitative research methods are being used in the process of developing personas, but their recognition as a research tool is more recent [2,3,7,9,10,12-14, 16,17,20,27,31-33]. Within the Smartline Research and Innovation Project, a mixed methods process was developed to create personas from survey, household sensor, and interview data. The Smartline Archetypes were created to facilitate innovation by making the project data more accessible, particularly to small and medium-sized enterprises working in sectors related to eHealth and eWell-being.

The process used to develop the Smartline Archetypes employed existing research methods, some of which, such as k-prototype cluster analysis, had previously been applied in persona development, whereas the qualitative approach and incorporation of environmental sensor data were novel. Holden et al [3] reported that they were able to “demonstrate the value of using largely qualitative data from a multiyear study but also identify the challenges of prolonged analysis and the difficulty of incorporating a rich and heterogeneous set of findings into a single design.” Therefore, the high level of triangulation we found between our data sources and the relatively rapid analytical methods applied to the qualitative data are significant developments. Overall, the approach was truly multidisciplinary, with contributions from epidemiology, health service research, mathematics, geography, and community engagement coming together into a product that reflects more than the sum of its parts [65]. Subsequently, it has been possible to apply the Smartline Archetypes in multiple ways with the project participants themselves and other stakeholders.

Data are crucial to research but can also be highly controversial, particularly with the new types and volumes of data that are becoming available. Calls for open science to increase transparency and accessibility of research meet the challenges of maintaining the duty of confidentiality regarding the data the public trusts to share with us [66]. Developing and maintaining trust in how participant data will be used is quite rightly recognized as fundamental to health research using data. Being transparent about how personal patient data are going to be used links to calls for great statistical literacy [67], which is supported by engaging communities in designing dissemination tools. The Smartline Archetypes provided an engaging opportunity to anonymously present the data collected by the study back to the participants and other stakeholders, overcoming some of the barriers to engaging with the data, such as statistical literacy.

### Limitations and Areas for Development

Despite these valuable uses identified for the Smartline Archetypes, we also identified a number of weaknesses or challenges in their development. The need to specify the number of clusters to be created by the k-prototype method might limit the use of such clustering methods. Although it is possible to base the number of clusters on the data, it might also be necessary to be pragmatic and specify a certain number of clusters, which would affect the validity of the methods applied. Clustering analyses make it possible to consider any number clusters and categorize the performance of the clustering accordingly (eg, via “elbow method”). This can accommodate or challenge prespecified requirements (eg, required minimum or maximum number of classes or clusters).

The development of personas could be based on stereotypical views of individuals or groups [5,30]. Basing our personas on the mathematical analysis of the survey data, the basic characteristics were derived using some objective criteria. Even in these circumstances, adding further embellishments to the personas could be influenced by unconscious biases or stereotypes. This influence could have occurred during the thematic analysis; however, by involving community partners and a team of researchers in this process, we hope that this risk has been minimized. This approach to persona development could also challenge stereotypes. For example, in this study, could some of the Smartline Archetypes be people who do not reflect stereotypes of social housing residents?

Starting with the survey data meant that the clusters identified emphasized the biases in the data set in terms of gender, age, ethnicity, etc. Community partners underlined that the data on which the Smartline Archetypes were based did not reflect the whole community, just those approached and willing to participate in the study. Subsequently, some diversity was added to the Smartline Archetypes, which might limit their validity. More extensive testing and validation of the Smartline Archetypes with the research participants and other stakeholders would be valuable but needs to be balanced against the risk of individual biases shaping the Archetypes. Archetype #7 “Sarah Jones” revealed a particular challenge to the use of quantitative methods alone to derive personas. Although the k-prototype methods grouped the people in this Smartline Archetype as being similar, the qualitative methods revealed significant differences in their circumstances. As the Smartline Archetype with the largest number of interview transcripts, the variation might simply reflect the larger volume of qualitative data or might reflect that objective data cannot adequately capture human experience and similar quantitative data might hide important differences between people. It is worth noting that all variables were equally weighted in the clustering process, but another approach could be to use different weightings to dictate the importance of certain characteristics over others. More theoretically, there is a need to consider whether the personas reflect collective fundamental but observable characteristics (archetypes) within which variation might be of interest or latent, hidden, or primordial ideas as in prototypes [20,27]. This distinction in the type of persona will depend upon the uses to which the personas will be put but might be an important distinction when comparing personas between studies or populations.

### Conclusions

Personas are a widely adopted tool that could prove useful in research, especially in using research to inform policy, practice, and business engagement. Methods are available to bring together various types of data into personas, and the resulting personas are recognized for being useful in communicating complex data [5]. The most appropriate methods to produce personas will depend on the specific application and data available, meaning that this approach is adaptable to a range of projects and disciplines. Unlike previous research, Smartline personas were created by layering quantitative survey, household sensor, and qualitative interview data, providing a novel multifaceted perspective. Personas were used within the Smartline project to maintain participant privacy while also increasing data accessibility. Therefore, the participants themselves were better able to engage with their own data and the project, and stakeholders from multiple sectors could use the project to inform innovation. Subsequently, personas represent an opportunity for broader engagement with research and greater policy and practice impact.

## Appendix

Multimedia Appendix 1 Plot of total sum of squared distances between cluster members and their respective cluster center.

Multimedia Appendix 2 Smartline social network analysis showing ego Smartline Archetype.

## Abbreviations

ANOVA: analysis of variance
PM_2.5_: atmospheric particulate matter that have a diameter of less than 2.5 μm
SME: small and medium-sized enterprise
