# Supplementary material for: Fostering Engagement With Health and Housing Innovation: Development of Participant Personas in a Social Housing Cohort
Source: JMIR Public Health Surveill. 2021 Feb 16;7(2):e25037. doi: 10.2196/25037 (PMC7925145; doi:10.2196/25037)

Original Paper

Fostering Engagement With Health and Housing Innovation: Development of Participant Personas in a Social Housing Cohort

Multimedia appendix 2. Smartline social network analysis showing ego Smartline Archetype

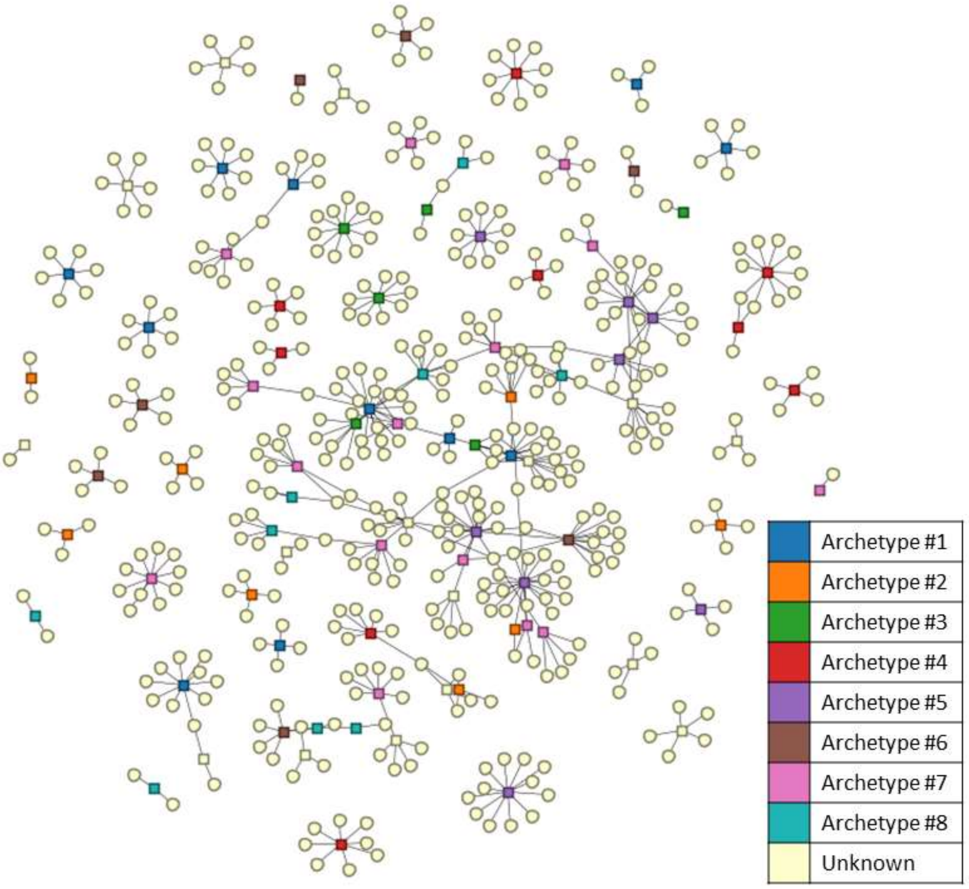

Supplement: Multimedia Appendix 2 [file publichealth_v7i2e25037_app2.pdf]
